# Infants display reduced NK cell responses in RSV and increased inflammatory responses in SARS-CoV-2 infections

**DOI:** 10.21203/rs.3.rs-5640872/v1

**Published:** 2025-01-13

**Authors:** Duygu Ucar, Asa Thibodeau, Asuncion Mejias, Djamel Nehar-Belaid, Radu Marches, Zhaohui Xu, Giray Eryilmaz, Steven Josefowicz, Silke Paust, Virginia Pascual, Jacques Banchereau, Octavio Ramilo

**Affiliations:** Jackson Laboratories; The Jackson Laboratory; Department of Infectious Diseases, St. Jude Children's Research Hospital; Jackson Laboratory; The Jackson Laboratory for Genomic Medicine; St. Jude Children's Research Hospital; The Jackson Laboratory; Weill Cornell Medicine; The Jackson Laboratory; Weill Cornell Medicine; Baylor Institute for Immunology Research; St. Jude Children's Research Hospital

## Abstract

Respiratory syncytial virus (RSV) is the leading cause of lower respiratory tract infection hospitalizations in infants and poses a significantly higher risk of respiratory failure than SARS-CoV-2. The mechanisms underlying these differences remain unclear. We analyzed blood samples from infants (median age 2.3 months) with SARS-CoV-2 (n = 30), RSV (n = 19), and healthy controls (n = 17) using single-cell transcriptomics and epigenomics, and cytokine profiling. Both viruses triggered comparable interferon responses across PBMC subsets but differed in NK cell and inflammatory responses. Severe RSV cases showed reduced NK cell frequencies, lower *IFNG* expression, and decreased chromatin accessibility at T-BET and EOMES binding sites. RSV infections were also associated with increased CD4^+^ T_EMRA_, memory T_reg_ and transitional B cells. In contrast, SARS-CoV-2 was characterized by stronger pro-inflammatory signatures, including increased NFKB pathway activity and higher serum TNF concentrations. These findings highlight distinct immune responses to RSV and SARS-CoV-2, providing insights that may inform clinical decisions.

## INTRODUCTION

Respiratory viral infections including those caused by respiratory syncytial virus (RSV) and severe acute respiratory syndrome coronavirus 2 (SARS-CoV-2) pose significant risks to infants^1–3^. Infants have developing immune systems that rely predominantly on innate immunity, along with smaller airways, making them more vulnerable to inflammation and severe respiratory morbidity^4^. RSV is a leading cause of lower respiratory tract infection, including bronchiolitis and pneumonia, leading to hospitalization in infants, and is the second leading cause of infant mortality globally^5–8^. Conversely, the clinical presentation of SARS-CoV-2 infections in infants tend to be milder, with lower morbidity and hospitalization rates compared to RSV^9^. This contrast between these two respiratory viruses was particularly evident during the early stages of the COVID-19 pandemic. The biological mechanisms behind these differences remain poorly understood. Dissecting the immune responses of infants to these two respiratory viruses may help explain the observed clinical disparities.

RSV infections can significantly impact infant immune cells, disrupting the normal functions of T cells and macrophages and impairing their ability to effectively control the infection^10^. Our previous studies uncovered blood-driven transcriptional signatures of RSV in children and infants^11,12^. Young children with mild disease had greater induction of interferon and plasma cell genes and decreased expression of inflammation and neutrophil genes compared to children with severe disease^11,12^. Severe RSV disease was associated with increased numbers of HLA-DR^low^ monocytes and reduced interferon responses^11–13^. Although these studies provided valuable insights into RSV disease signatures, bulk profiling lacked the resolution needed to identify cell-type-specific immune responses driving these signatures. Single-cell profiling overcomes this limitation, enabling the characterization of immune signatures at the cellular level.

Little is known about how SARS-CoV-2 virus interacts with the developing immune systems of infants. Infants with mild SARS-CoV-2-infection developed robust antibody responses, which persisted for months^14^. The nasal mucosal immune response in these infants was characterized by the presence of inflammatory cytokines, IFNα, and markers for Th17 cells and neutrophils^14^. In contrast, the immune response in the blood showed activation of innate cells and expression of ISGs, but without notable inflammation^14^. We recently observed significant changes in immune cell compositions in infants with moderate and severe SARS-CoV-2 infection, with most cell types displaying increased interferon-stimulated gene (ISG) expression^15^. Compared to adults, infants exhibited a similar ISG response in monocytes but a more pronounced ISG response in T and B cells, highlighting unique aspects of early-life immunity to SARS-CoV-2^15^. While interferon activation has been documented in both RSV and SARS-CoV-2 infections in infants^11,12,14^, the extent to which these responses are comparable and the roles of other immune pathways, such as inflammation, remain unclear. Furthermore, while epigenetic mechanisms are critical in shaping immune responses to infectious diseases, their role in RSV and SARS-CoV-2 pathogenesis, particularly in infants, is not well understood.

To address these gaps, we recruited two cohorts of infants (median age: 2.3 months) infected with RSV (n = 30) or SARS-COV-2 (n = 19) across varying disease severities (mild, moderate, severe), alongside age-matched healthy controls (n = 17). We profiled peripheral blood mononuclear cells (PBMCs) using single cell RNA-sequencing (scRNA-seq) and single nucleus assay for transposase-accessible chromatin with sequencing (snATAC-seq) to investigate transcriptional and epigenetic responses.

## RESULTS

### Study design

**Figure 1:**
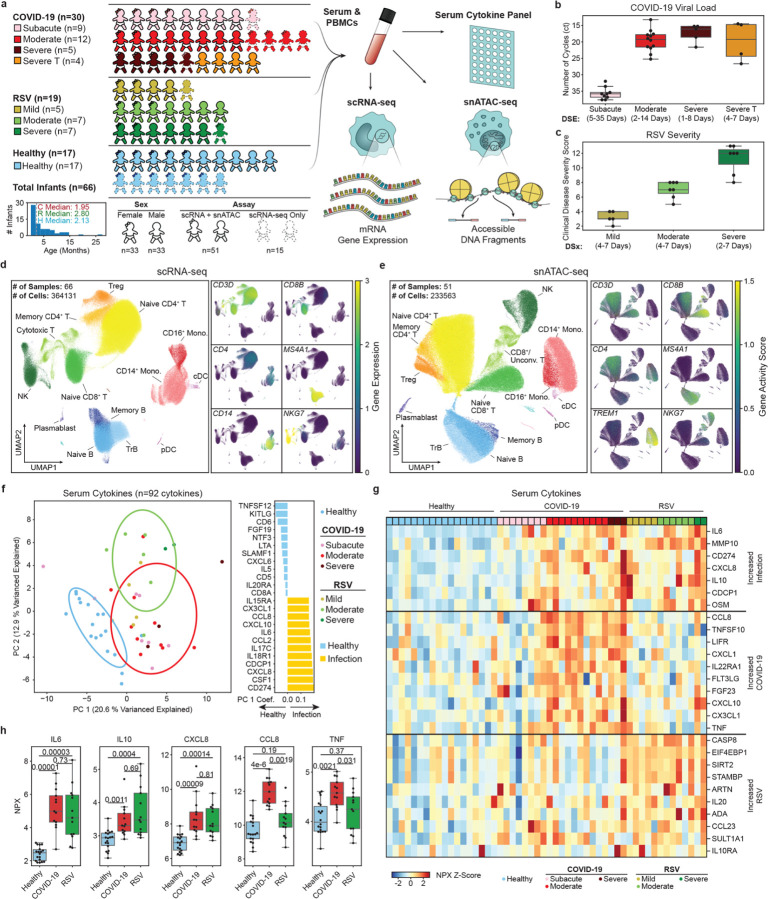
Study design and data overview. **a**, Summary of study design. SARS-CoV-2 and RSV sample numbers by disease severity and dexamethasone treatment. Histogram shows age distribution for all infants with median ages for healthy, COVID-19 (moderate and severe) and RSV (mild, moderate, and severe). PBMCs were sequenced using scRNA-seq and snATAC-seq. Serum samples were profiled using Olink 96 inflammation panel. **b**, Viral loads determined by the number of cycles (ct) required to detect SARS-CoV-2. **c**, RSV disease severity determined by an established clinical disease severity score (CDSS). **d**,**e**, UMAPs displaying all PBMCs for scRNA-seq and snATAC-seq modalities with respective cell lineage markers: gene expression for scRNA-seq and gene activity scores for snATAC-seq. **f**, (left) Scatterplot of the first two principle components of 92 serum cytokines. (right) Top and bottom cytokines showing the most significant coefficients contributing to PC1. **g**, Heatmap of the top increasing serum cytokine concentrations in infected infants. **h**, Boxplots of select cytokines showing specific examples of shared and SARS-CoV-2 specific increases in proinflammatory cytokines. Adjusted p-values in **h** were calculated using Dunn’s test for multiple comparisons.

From February 2019 through January 2022, we enrolled 49 infants and young children hospitalized with SARS-CoV-2 (n = 30; median IQR age: 1.9 [0.9–7.7] months) or RSV infection (n = 19; 2.8 [1.5–5.4] months) and obtained blood samples within 24 hours for peripheral blood mononuclear cell (PBMC) isolation and serum proteome analyses. We also included samples from a cohort of healthy controls (n = 17; 2.1 [1.8–8.4] months) enrolled before the COVID-19 pandemic. The cohorts were age- and sex-matched with the majority (89%) of infants being less than 12 months old (Fig. 1a, Extended Data Fig. 1a–c). The basic demographic and clinical characteristics of the 66 infants included in this study are summarized in Table S1.

Infants with COVID-19 were classified as subacute (n = 9); moderate (n = 12), or severe (n = 9) based on three criteria: (1) level of clinical care (oxygen administration, PICU care); (2) SARS-CoV-2 viral loads at admission, and (3) days post-exposure to an index COVID-19 case (Fig. 1b; Table S1). Infants in the subacute COVID-19 group had low viral loads and longer duration since exposure, with a median of 21 days. Children with moderate COVID-19 had high SARS-CoV-2 loads and were all hospitalized in the inpatient ward with respiratory tract symptoms and/or fever. Most (90%) infants in the severe COVID-19 group required PICU care for lower respiratory tract disease. Four of these infants were treated with dexamethasone, and classified as severe treated (referred to as “severe T”) to account for steroid effects on immune responses^16,17^. All infants with acute RSV infection presented with respiratory symptoms and were classified as mild (n = 5), moderate (n = 7), or severe (n = 7) based on a standardized clinical disease severity score^11,18,19^ (Fig. 1c).

PBMCs were isolated and profiled using scRNA-seq (66 samples) and snATAC-seq (51 samples). After quality control and batch correction (Table S2, Extended Data Fig. 1d,e), scRNA-seq and snATAC-seq data yielded 364,131 and 233,563 cells, respectively (Fig. 1d,e). Major immune cell lineages were identified using marker gene expression and gene activity scores (Fig. 1d,e, Extended Data Fig. 1f). Detailed clustering and annotation within these lineages identified 48 distinct subsets in scRNA-seq (Extended Data Fig. 1f), of which 26 were also defined in the snATAC-seq dataset through label transfer (Extended Data Fig. 1g). Additionally, serum cytokines were analyzed using the Olink 96 Inflammation panel (n = 54 samples, including 37 infants from this cohort and 17 additional healthy controls) (Fig. 1a, Table S3).

### Pro-Inflammatory cytokine responses differ between SARS-CoV-2 and RSV infections

Principal component analysis (PCA) of cytokine concentrations revealed clear distinctions in infants based on infection status (PC1) and infection type (PC2) (Fig. 1f). Key cytokines involved in inflammatory modulation, including IL-6, IL-10, and CXCL8 (IL-8), were elevated to comparable concentrations in both infections compared to healthy controls (p_adj_ < 0.0011) (Fig. 1g,h). IL-6 concentrations also correlated with RSV clinical disease severity scores (Pearson r = 0.7, p_adj_ = 0.0075; Extended Data Fig. 1h), consistent with our previous observations^19^. Virus-specific differences were also noted: SARS-CoV-2-infected infants exhibited increased concentrations of TNF, CCL8, and CXCL1 (Fig. 1g,h), whereas RSV-infected infants displayed increased levels of CASP8 (Fig. 1g), a molecule involved in initiating apoptosis and modulating inflammasome^20^. These findings highlight both shared and unique cytokine responses in SARS-CoV-2 and RSV infections.

### Myeloid cells mount strong interferon transcriptional responses in both infections

**Figure 2:**
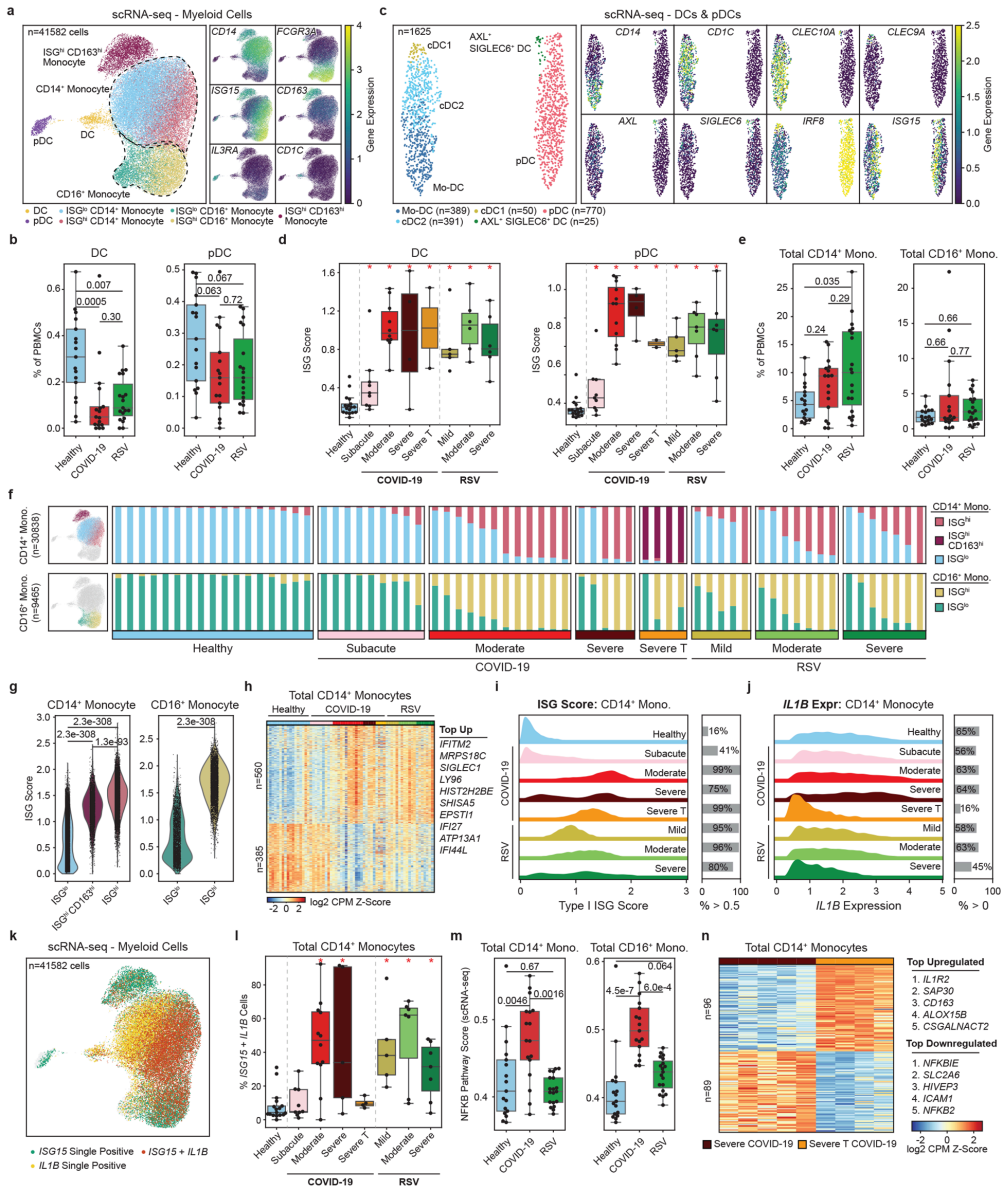
Reduced DC and pDC frequencies and upregulation of ISGs in myeloid cells. **a**, UMAP of myeloid cells with marker genes describing CD14^+^ and CD16^+^ monocytes (*CD14* and *FCGR3A*), CD163^hi^ monocytes (*CD163*), DCs (*CD1C*) and pDCs (*IL3RA*). Monocyte clusters separated into ISG^hi^ (*ISG15*) and ISG^lo^ states. **b**, Pairwise comparisons of total DC and pDC frequencies as a percentage of total PBMCs. **c**, Clustering of DCs and pDCs describing Mo-DCs (*CD14*), cDC1s (*CLEC9A*), cDC2s (*CLEC10A*) and AXL+ SIGLEC6+ DCs (*AXL* and *SIGLEC6*). **d**, Healthy control comparisons of ISG scores with clinical groups. **e**, Pairwise comparisons of total CD14^+^ monocyte and total CD16^+^ monocyte frequencies as a percentage of total PBMCs. **f**, Relative frequencies of CD14^+^ monocyte and CD16^+^ monocyte for ISG^hi^, ISG^lo^, and CD163^hi^ cell states. **g**, Pairwise comparisons of ISG scores for CD14^+^ and CD16^+^ monocyte with respect to cell state. **h**, Heatmap of combined healthy vs COVID-19 (moderate and severe) and healthy vs RSV (mild, moderate and severe) differentially expressed genes and top differentially expressed genes in CD14^+^ monocytes. **i**,**j**, Ridge plots of ISG scores and *IL1B* expression in CD14+ monocytes for all clinical groups. **k**, UMAP of cells expressing or co-expressing *ISG15* and/or *IL1B*. **l**, Healthy control comparisons of CD14+ monocytes co-expressing *ISG15* and *IL1B* as a percent of total CD14^+^ monocytes. **m**, Pairwise comparisons of NFKB pathway scores for total CD14^+^ and CD16^+^ monocytes. **n**, Heatmap of differentially expressed genes in CD14^+^ monocytes between severe and severe T COVID-19 infant groups. Statistical tests for all pairwise comparisons between healthy controls, COVID-19 (moderate and severe untreated) and RSV (mild, moderate and severe) and monocyte subsets were calculated using Dunn’s test of multiple comparisons (panels **b**,**e**,**g**,**m**). Statistical tests for healthy control comparisons across clinical groups were calculated using Mann-Whitney rank sum tests followed by Benjamini Hochberg multiple hypothesis correction (panels d,l). Significant comparisons to healthy controls (p_adj_ < 0.05) are indicated by a red asterisk.

Myeloid cells were annotated into seven subsets: ISG^hi^ and ISG^lo^ CD14^+^ monocytes (n = 9,086, n = 17,730), ISG^hi^ and ISG^lo^ CD16^+^ monocytes (n = 5,569, n = 3,772), ISG^hi^ CD163^+^ monocytes (n = 3,800), dendritic cells (DCs) (n = 855), and plasmacytoid dendritic cells (pDCs) (n = 770) (Fig. 2a). The frequencies of DCs and pDCs were lower (p_adj_ < 0.007 for DCs, p_adj_ < 0.067 for pDCs) in both infections compared to healthy controls (Fig. 2b, Extended Data Fig. 2a).

DC clustered into monocyte-derived DCs (mo-DCs) (*CD14* expression, n = 389), conventional DC type-2 (cDC2) (*CD1C*, n = 391), cDC type-1 (cDC1) (*CLEC9A*, n = 50), and AXL^+^ SIGLEC6^+^ DCs (*AXL* and *SIGLEC6*, n = 25) (Fig. 2c). Mo-DCs, cDC1s and AXL^+^ SIGLEC6^+^ DCs were less frequent (p_adj_ < 0.051) in both infections, while cDC2 frequency was lower (p_adj_ = 0.042) only in SARS-CoV-2 infection (Extended Data Fig. 2b). High ISG (e.g., *ISG15*) expression was observed in infected infants (Extended Data Fig. 2c). Cumulative transcriptional ISG scores confirmed significantly higher ISG expression in infected infants (p_adj_ < 0.001; Extended Data Fig. 2d, Table S4).

CD14^+^ monocytes were more frequent in RSV-infected infants compared to healthy controls (p_adj_ = 0.035; Fig. 2e, Extended Data Fig. 2a). Three ISG^hi^ subsets were identified within monocytes: ISG^hi^ CD14^+^ monocytes, ISG^hi^ CD163^hi^ CD14^+^ monocytes, and ISG^hi^ CD16^+^ monocytes. These ISG^hi^ clusters were more frequent in acute infections but not in the subacute COVID-19 group (Fig. 2f, Extended Data Fig. 2e). The ISG^hi^ CD163^hi^ CD14^+^ monocyte subset was exclusively found in four dexamethasone-treated infants with severe COVID-19 (severe T, Fig. 2f). Although these cells retained ISG expression, levels were lower than those in ISG^hi^ CD14 + monocytes, suggesting that dexamethasone attenuates interferon responses in monocytes (Fig. 2g).

Differential gene expression analyses revealed 560 and 601 upregulated genes in total CD14^+^ and CD16^+^ monocytes, respectively, including ISGs (e.g., *IFI44*, *IFI44L*, *IFI27*, *IFITM2*, and *OASL*) and inflammatory molecules (Fig. 2h, Extended Data Fig. 2f–h, Table S5). Downregulated genes were enriched in pathways related to protein synthesis and transcription (Extended Data Fig. 2g–h). ISG scores were consistently high across acute infections, with no significant differences between the two diseases or among disease severities (Fig. 2i, Extended Data Fig. 2i). Together, these results demonstrate that both SARS-CoV-2 and RSV infections induce robust interferon transcriptional responses in DCs, pDCs, and monocytes, regardless of disease severity.

### Lower IL1B expression in severe RSV and higher NFKB gene expression in COVID-19

To investigate pro-inflammatory signatures, we first examined *IL1B* expression. In CD14 + monocytes, *IL1B* was lower in RSV-infected infants compared to healthy controls (p_adj_ = 0.046), with the decrease primarily driven by infants with severe RSV disease (p_adj_ = 0.005, Fig. 2j, Extended Data Fig. 2j). CD14^+^ monocytes co-expressing *ISG15* and *IL1B* were more frequent in infected infants, regardless of disease severity (Fig. 2k,l).

Next, we analyzed the NF-κB pathway genes (Table S4), which were upregulated in SARS-CoV-2-infected infants compared to healthy controls (p_adj_ = 0.0046 for CD14^+^ monocytes and p_adj_ < 0.001 for CD16^+^ monocytes) and RSV-infected infants (p_adj_ = 0.0016 for CD14^+^ monocytes and p_adj_ < 0.001 for CD16^+^ monocytes) (Fig. 2m, Extended Data Fig. 2k). Genes within this pathway, including *IL1A* and *TNF*, were more expressed in moderate and severe COVID-19 cases compared to RSV (Extended Data Fig. 2l–m). Steroid treatment modulated these inflammatory responses. Both *IL1B* expression and NF-κB pathway scores were reduced in steroid-treated (severe T) infants (Extended Data Fig. 2j,k). To further explore these effects, we compared the transcriptomes of CD14^+^ monocytes from severe and severe T COVID-19 cases (Table S5). In the treated infants, *NFKBIE* and *NFKB2* were downregulated, while *CD163* and *IL1R2* were upregulated (Fig. 2n), suggesting a shift toward an anti-inflammatory state.

In summary, SARS-CoV-2 and RSV induced distinct inflammatory profiles in monocytes. *IL1B* expression was downregulated in infants with severe RSV infection, whereas NF-κB pathway genes were upregulated with SARS-CoV-2 infection.

### Durable epigenetic ISG signatures in infant monocytes

**Figure 3:**
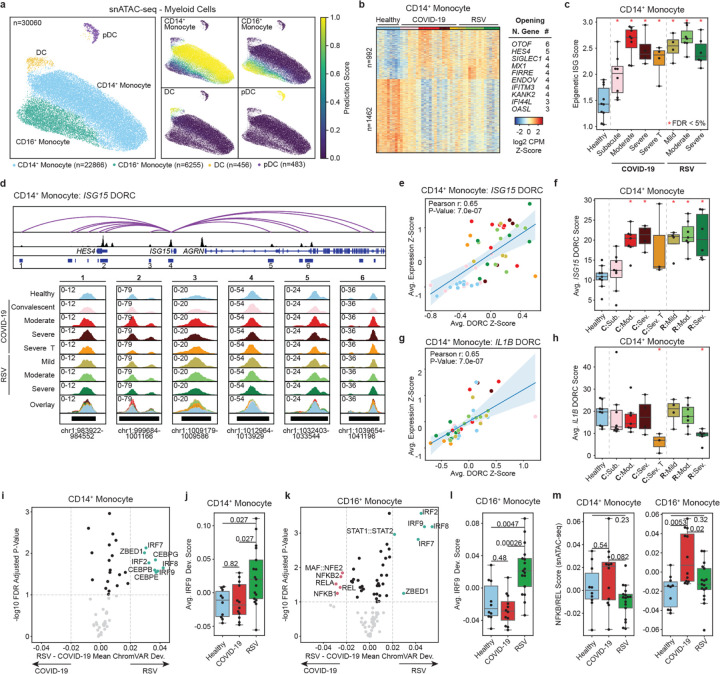
Chromatin remodeling of ISGs and IRFs in monocytes. **a**, UMAP of myeloid subsets in snATAC-seq with label transfer prediction scores from scRNA-seq annotations. **b**, Heatmap of combined healthy vs COVID-19 (moderate and severe) and healthy vs RSV (mild, moderate and severe) differentially accessible peaks alongside the genes with the greatest number of nearby peaks in CD14^+^ monocytes. **c**, Epigenetic ISG scores based on opening peaks near ISGs in CD14^+^ monocytes. **d**, Genome browser example of *ISG15* DORC. Arcs indicate significant correlations between accessibility and *ISG15* expression. **e**, Pearson correlation of *ISG15* DORC z-scores and *ISG15* expression z-scores. **f**, Healthy control comparisons of *ISG15* DORC scores across clinical groups. **g**, Pearson correlation of *IL1B* DORC z-scores and *IL1B* expression z-scores. **h**, Healthy control comparisons of *IL1B* DORC z-scores across clinical groups. **i**, Volcano plot of significant transcription factors comparing COVID-19 (moderate and severe) and RSV (mild, moderate and severe) groups in CD14^+^ monocytes. **j**, Pairwise comparisons of IRF9 chromVAR deviation scores in CD14^+^ monocytes. **k**, Volcano plot of significant transcription factors comparing COVID-19 (moderate and severe) and RSV (mild, moderate and severe) groups in CD16^+^ monocytes. **l**, Pairwise comparisons of IRF9 chromVAR deviation scores in CD16^+^ monocytes. **m**, Pairwise comparisons of NFKB/REL scores derived from chromVAR deviations. Statistical tests for pairwise comparisons between healthy controls, COVID-19 (moderate and severe) and RSV (mild, moderate and severe) were calculated using Dunn’s test of multiple comparisons (panels **j**,**l**,**m**). Statistical tests for healthy control comparisons across clinical groups were calculated using Mann-Whitney rank sum tests followed by Benjamini Hochberg multiple hypothesis correction (panels **c**,**f**,**h**). Significant comparisons to healthy controls (p_adj_ < 0.05) are indicated by a red asterisk.

We investigated the epigenomes of CD14^+^ (n = 22,866 cells) and CD16^+^ monocytes (n = 6,255) in both infections (Fig. 3a). In CD14^+^ monocytes, we identified 2,454 differentially accessible peaks (992 opening, 1,462 closing) between healthy and infected infants (Fig. 3b). Similarly, in CD16^+^ monocytes, we identified 1,122 differentially accessible peaks (411 opening, 711 closing; Extended Data Fig. 3a). Opening peaks were annotated to multiple ISGs (e.g., *MX1, IFITM3, and IFI44L*) and enriched for interferon modules (Fig. 3b, Extended Data Fig. 3a,b, Table S6).

We calculated an epigenetic ISG score based on chromatin accessibility levels at ISG loci in monocytes (Fig. 3c, Extended Fig. 3c). This score was higher in all infected infants, including those in the subacute COVID-19 group (p_adj_ < 0.014, Fig. 3c). To assess the persistence of this epigenetic interferon activity, we reanalyzed ATAC-seq profiles of CD14 + monocytes from convalescent adults 2–4 months post-SARS-CoV-2 infection^21^. Using infant-derived ATAC-seq peaks, we found that epigenetic interferon activity persisted in adults, with higher scores observed in severe COVID-19 cases compared to healthy controls (p_adj_ < 0.001) and mild COVID-19 cases (p_adj_ = 0.101; Extended Data Fig. 3d). These results suggest that SARS-CoV-2 induces durable epigenetic ISG signatures in monocytes in both infants and adults.

To further link chromatin accessibility changes to gene expression, we performed Domains of Regulatory Chromatin (DORC) analysis^22^ (Table S7). *ISG15* was associated with 13 peaks that cumulatively correlated with its expression (Pearson *r* = 0.65, *p* = 7.0e-7; Fig. 3d,e). DORC scores for *ISG15* were elevated (p_adj_ < 0.008) in acute cases of both infections (Fig. 3f). In contrast, *IL1B* DORC scores were reduced in steroid-treated severe COVID-19 cases and severe RSV cases (p_adj_ = 0.032 and 0.006, respectively; Fig. 3g–h).

To study changes in transcription factor (TF) binding activity, we used chromVAR^23^ (Fig. 3i–m, Extended Data Fig. 3e–h). Binding sites for interferon regulatory factors (IRF) were more accessible in RSV-infected infants compared to SARS-CoV-2-infected infants in both CD14^+^ and CD16^+^ monocytes (Fig. 3i–l, Extended Data Fig. 3e–g). Despite this, IRF genes were upregulated in response to both SARS-CoV-2 and RSV infections (Extended Data Fig. 3i,j). Conversely, binding sites for NF-κB family factors were more accessible in SARS-CoV-2-infected infants, particularly in CD16^+^ monocytes (Fig. 3m, Extended Data Fig. 3h).

In summary, we observed a persistent epigenetic interferon response in infant monocytes that extended beyond transcriptional changes. Virus-specific differences were evident in TF binding site accessibility, with increased IRF accessibility in RSV and increased NF-κB/REL factor accessibility in SARS-CoV-2.

### Reduced NK cell frequencies and downregulation of IFNG/IFNG-AS1 in severe RSV

**Figure 4:**
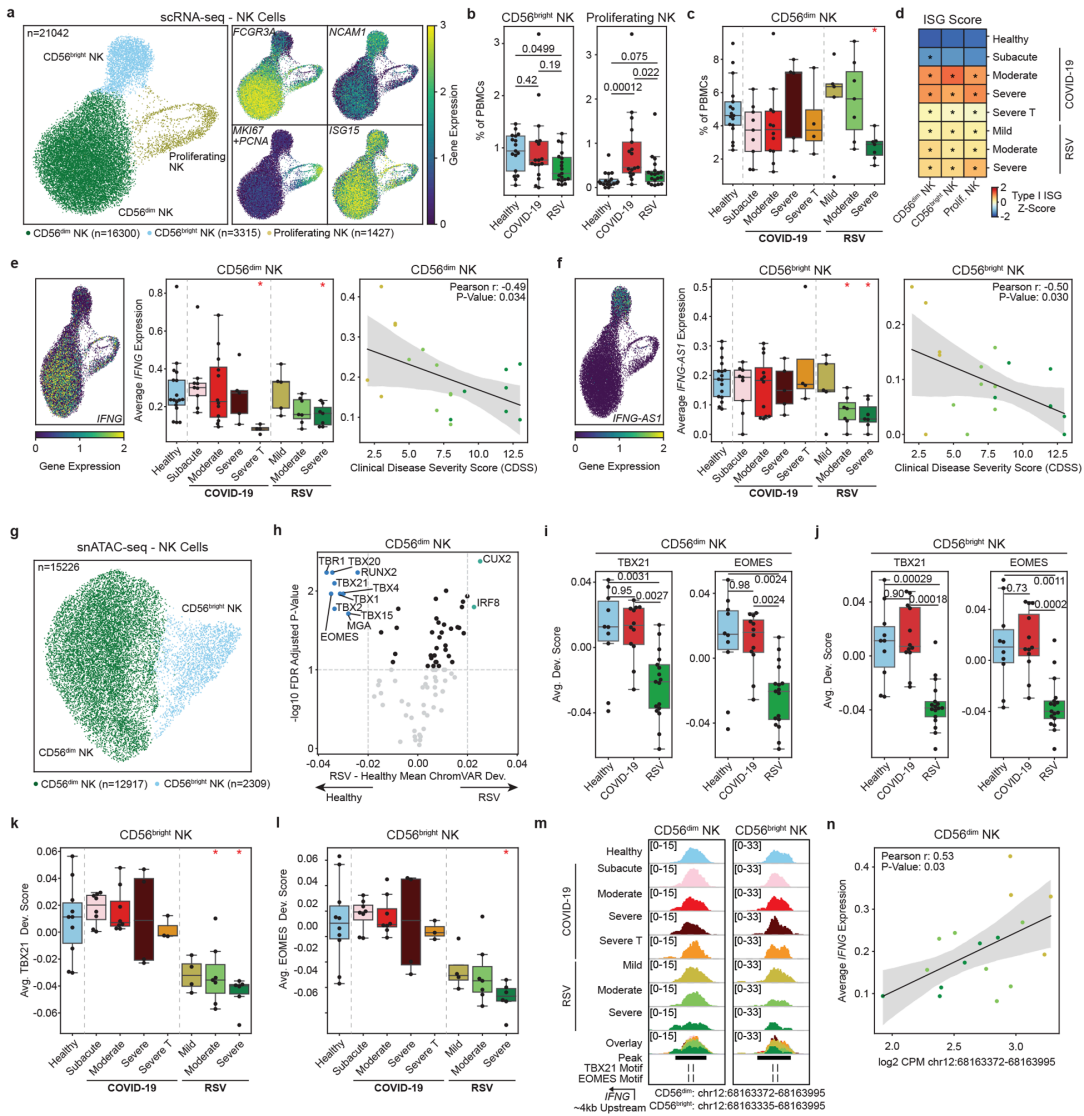
Reduced NK frequencies, *IFNG* expression, and TBET/EOMES binding site accessibility in RSV. **a**, UMAP of NK cells and marker genes describing CD56^dim^ NK (*FCGR3A* and low *NCAM1*), CD56^bright^ NK (*NCAM1*), and proliferating NK (*MKI67*). **b**, Pairwise comparisons of CD56^bright^ and proliferating NK cell frequencies as a percentage of total PBMCs. **c**, Healthy control comparisons to clinical groups for CD56^dim^ NK cell frequencies. **d**, Heatmap of ISG scores for NK cell populations and clinical groups. Asterisks denote significant differences with healthy controls. **e**,**f**, (left) UMAPs of *IFNG*/*IFNG-AS1* expression. (center) Healthy control comparisons to clinical groups for *IFNG*/*IFNG-AS1* expression in CD56^dim^ NK/CD56^bright^ NK cells. (right) Pearson correlation of *IFNG* expression and *IFNG-AS1* expression with clinical disease severity scores. **g**, UMAP of NK subsets in snATAC-seq with prediction scores from label transfer with scRNA-seq annotation. **h**, Volcano plot of statistically significant transcription factors comparing infants with RSV (mild, moderate and severe) to healthy controls in CD56^dim^ NK cells. **i**,**j**, Pairwise comparisons of TBX21 and EOMES chromVAR deviation scores for CD56^dim^ NK/CD56^bright^ NK cells. **k**,**l**, Healthy control comparisons of TBX21 and EOMES chromVAR deviation scores for CD56^bright^ NK cells. **m**, Genome browser examples of a peak 4kb upstream of *IFNG* harboring motifs for TBX21 and EOMES in CD56^dim^ and CD56^bright^ NK cells for each clinical group. **n**, Pearson correlation of *IFNG* expression in CD56^dim^ NK cells and peak (panel **m**) accessibility for infants infected with RSV. Scatter plot colors correspond to RSV disease severity in **m**. Statistical tests for pairwise comparisons between healthy controls, COVID-19 (moderate and severe) and RSV (mild, moderate and severe) were calculated using Dunn’s test of multiple comparisons (panels **b**,**i**,**j**). Statistical tests for healthy control comparisons across clinical groups were calculated using Mann-Whitney rank sum tests followed by Benjamini Hochberg multiple hypothesis correction (panels **c**,**e**,**f**,**k**,**l**). Significant comparisons (p_adj_ < 0.05) to healthy controls are indicated by a red asterisk.

We identified three NK cell subsets: mature CD56^dim^ (*FCGR3A*, n = 16,300), immature CD56^bright^ (*NCAM1*, n = 3,315), and proliferating NK cells (*MKI67 and PCNA*, n = 1,427) (Fig. 4a). The frequency of CD56^bright^ NK cells was lower (p_adj_ = 0.0499) in all RSV-infected infants compared to healthy controls, while the frequency of CD56^dim^ NK cells was lower (p_adj_ = 0.01) specifically in severe RSV cases (Fig. 4b,c, Extended Data Fig. 4a,b). Conversely, the frequency of proliferating NK cells was increased in SARS-CoV-2 infected infants (Fig. 4b, Extended Data Fig. 4b). *ITGA4*, a marker for lung migration, was upregulated in RSV-infected infants across all NK cell subsets (Extended Data Fig. 4cd), suggesting enhanced NK cell recruitment to the lungs with RSV.

ISG scores were higher across all NK subsets in infants with acute SARS-CoV-2 or RSV infections (p_adj_ < 0.0044, Fig. 4d, Extended Data Fig. 4e–g). Cytotoxic genes, *GZMB* and *PRF1*, were also upregulated in both infections within CD56^dim^ and CD56^bright^ NK cells (Extended Data Fig. 4h). NK cells produce interferon gamma (IFN-γ) encoded by the *IFNG* gene, which is regulated by the long noncoding RNA *IFNG-AS1*^24^. *IFNG*, expressed primarily in CD56^dim^ NK cells, was downregulated in severe RSV and steroid-treated severe COVID-19 (p_adj_ = 0.019; Fig. 4e). *IFNG-AS1*, expressed primarily in CD56^bright^ NK cells, was downregulated in moderate and severe RSV cases (p_adj_ < 0.004, Fig. 4f). Expression levels of *IFNG* and *IFNG-AS1* inversely correlated with RSV disease severity, with lower levels observed in infants with more severe disease (Pearson r=-0.49 and − 0.50, p = 0.034 and 0.30 for CD56^dim^ and CD56^bright^ NK cells respectively; Fig. 4e,f). In summary, severe RSV infection is associated with reduced frequencies of NK cells and downregulated *IFNG* expression, highlighting impaired NK cell responses in these cases.

### T-BET and EOMES binding site accessibility is associated with IFNG expression in NK cells

We captured CD56^dim^ NK (n = 12,917) and CD56^bright^ NK (n = 2,309) subsets in snATAC-seq data (Fig. 4g). ChromVAR analysis revealed increased IRF binding site accessibility in both NK subsets for both infections (Table S8). However, an RSV-specific decline in chromatin accessibility at T-box family TF binding sites, including key NK cell maturation and IFN-γ production regulators TBX21 (T-BET) and EOMES^25^, was observed in both NK subsets (Fig. 4h,i,j, Extended Data Fig. 5a,b,c). This decline was more pronounced in moderate and severe RSV cases, particularly in CD56^bright^ NK cells (p_adj_ < 0.011, Fig. 4k,l,). Chromatin accessibility at EOMES binding sites was inversely correlated with RSV disease severity, with the lowest accessibility observed in infants with the most severe disease (Extended Data Fig. 5d,e). Notably, these reductions occurred independently of *TBX21* and *EOMES* expression levels (Extended Data Fig. 5f–g).

**Figure 5:**
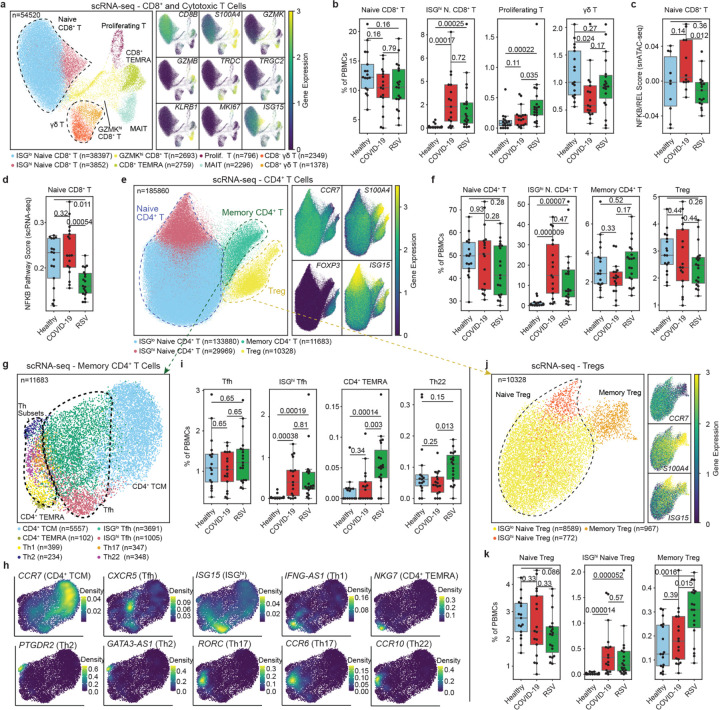
T cell alterations in infants infected with SARS-CoV-2 and RSV. **a**, UMAP of CD8^+^ and cytotoxic T cells with marker genes describing ISG^lo^ and ISG^hi^ (*ISG15*) naive CD8^+^ T, GZMK^hi^ CD8^+^ T (*GZMK*), CD8^+^ T_EMRA_ (*GZMB*), proliferating T (*MKI67*), MAIT (*KLRB1*), CD8^−^ γδ T (*TRDC* and *TRGC2*) and CD8^+^ γδ T (*CD8B*) cells. **b**, Pairwise comparisons of naive CD8^+^ T, ISG^hi^ CD8^+^ T, proliferating T, and γδ T cell frequencies. **c**, Pairwise comparisons of NFKB/REL scores from snATAC-seq data in naive CD8^+^ T cells. **d**, Pairwise comparisons of NFKB pathway scores from scRNA-seq data in naive CD8^+^ T cells. **e**, UMAP of CD4+ T cells with marker genes describing ISG^lo^ and ISG^hi^ (*ISG15*) naive CD4^+^ T, memory CD4^+^ T (*S100A4*) and T_reg_(*FOXP3*) cells. **f**, Pairwise comparison of naive CD4^+^ T, ISG^hi^ naive CD4^+^ T, memory CD4^+^ T, and T_reg_ cell frequencies. **g**, UMAP of memory CD4^+^ T clusters. **h**, Density plots of memory CD4^+^ T cluster marker genes. **i**, Pairwise comparisons of Tfh, ISG^hi^ Tfh, CD4^+^ TEMRA and Th22 cell frequencies. **j**, UMAP of T_reg_ cells with marker genes describing ISG^lo^ and ISG^hi^ (*ISG15*) naive T_reg_, and memory T_reg_ (*S100A4*) cells. **k**, Pairwise comparison of naive Treg, ISG^hi^ Treg and memory Treg cell frequencies. Statistical tests for pairwise comparisons between healthy controls, COVID-19 (moderate and severe) and RSV (mild, moderate and severe) were calculated using Dunn’s test of multiple comparisons (panels **b**,**c**,**d**,**f**,**i**,**k**).

A regulatory ATAC-seq peak containing T-BET and EOMES motifs, located 4kb upstream of the *IFNG* transcription start site (TSS), was accessible in both NK subsets (Fig. 4m). Chromatin accessibility at this locus inversely correlated with RSV disease severity in CD56^dim^ NK cells (Pearson *r* = –0.49, p_adj_ < 0.044; Extended Data Fig. 5h), with the lowest accessibility observed in the most severe cases (Fig. 5m, Extended Data Fig. 5i). Additionally, accessibility of this locus was positively correlated with *IFNG* and *IFNG-AS1* expression in both NK subsets (Fig. 4n, Extended Data Fig. 5j). These findings suggest that RSV disease severity is linked to impaired accessibility at regulatory loci critical for *IFNG* expression in NK cells.

### ISGs are upregulated in CD8^+^/cytotoxic T cells in both SARS-CoV-2and RSV

CD8^+^/cytotoxic T cells clustered into eight subsets: ISG^lo^ naive CD8^+^ T (n = 38,397), ISG^hi^ naive CD8^+^ T (*ISG15*, n = 3,852), GZMK^hi^ CD8^+^ T (*GZMK*, n = 2,693), CD8^+^ terminal effector memory T (T_EMRA_) (*GZMB*, n = 2,759), mucosal-associated invariant T (*KLRB1*, MAIT) (n = 2,296), proliferating T cells (*MKI67*, n = 796), γδ T (*TRDC* and *TRGC2*, n = 2,349), and CD8^+^ γδ T (n = 1,378) (Fig. 5a, Extended Data Fig. 6a). The frequency of ISG^hi^ naive CD8^+^ T cells was higher in RSV or SARS-CoV-2 infected infants (p_adj_ < 0.001, Fig. 5b, Extended Data Fig. 6b,c). ISG scores were increased across all subsets during acute disease (Extended Data Fig. 6d–j). However, no significant differences in ISGs were observed between RSV and SARS-CoV-2 or across disease severities (Table S5). The frequency of proliferating T cells, both CD8^+^ and CD4^+^, was higher in RSV-infected infants (p_adj_ < 0.021 compared to controls; p_adj_ < 0.064 compared to COVID-19; Extended Data Fig. 6c,k,l). The frequency of γδ T cells was lower in SARS-CoV-2 (p_adj_ = 0.024, Fig. 5b, Extended Data Fig. 6c), consistent with reports from SARS-CoV-2-infected adults and children^26–28^.

The most notable epigenetic differences between RSV and SARS-CoV-2 were observed in naive CD8^+^ T cells (Extended Data Fig. 7a,b). In these cells, binding sites for AP-1 transcription factors (e.g., FOS, FOSL2, and JDP2) and NF-κB member REL were more accessible in SARS-CoV-2-infected infants compared to RSV-infected infants (Extended Data Fig. 7b). NF-κB pathway genes were both upregulated and more accessible in SARS-CoV-2-infected infants compared to RSV-infected infants (Fig. 5c,d, Extended Data Fig. 7c,d).

In summary, infants mount robust interferon responses in CD8^+^/cytotoxic T cells in RSV and SARS-CoV-2 infections. However, SARS-CoV-2-infected infants showed greater chromatin accessibility and expression of pro-inflammatory NF-κB and AP-1 factors.

### RSV-infected infants have increased CD4^+^ T_EMRA_ and memory T_reg_ cells

CD4^+^ T cells clustered into four subsets: ISG^lo^ naive CD4^+^ T (*CCR7* 133,880) and ISG^hi^ naive CD4^+^ T (*ISG15*, n = 29,969), memory CD4^+^ T (*S100A4*, n = 14,048), and T_reg_ (*FOXP3*, n = 10,328) cells (Fig. 5e). While the overall frequency of naive CD4^+^ T cells did not change with infections, the frequency of ISG^hi^ naive CD4^+^ T cells increased in infected infants (p_adj_ < 0.001, Fig. 5f). No differences were observed in memory CD4^+^ T and T_reg_ cell frequencies (Fig. 5f).

Memory CD4^+^ T cells further clustered into eight subsets: CD4^+^ central memory T (TCM) (*CCR7*, n = 5,557), CD4^+^ T_EMRA_ (*NKG7*, n = 102), ISG^lo^ T follicular helper-like (Tfh) (*CXCR5*, n = 3,691), ISG^hi^ Tfh (*ISG15*, n = 1,005), Th1 (*IFNG-AS1*, n = 399), Th2 (*GATA3-AS1*, n = 234), Th17 (*RORC*, n = 347), and Th22 (*CCR10*, n = 348) cells (Fig. 5g,h, Extended Data Fig. 7e–g). The total Tfh cell frequencies were comparable across the three clinical groups, however, ISG^hi^ Tfh-like cells were increased in both infections (p_adj_ < 0.001, Fig. 5i, Extended Data Fig. 7h,i). CD4^+^; TEMRA cell frequencies were higher in RSV-infected infants (p_adj_ < 0.003, Fig. 5i, Extended Data Fig. 7i). Th22-like cell frequencies were higher in RSV-infected infants compared to SARS-CoV-2-infected infants (p_adj_ = 0.013, Fig. 5i).

T_reg_ cells clustered into three subsets: ISG^lo^ naive T_reg_ (*CCR7*, n = 8,589), ISG^hi^ naive T_reg_ (*ISG15*, n = 772), and memory T_reg_ cells (*S100A4*, n = 967) (Fig. 5j). While total naive T_reg_ frequencies remained unchanged, ISG^hi^ T_reg_ cells were more frequent in infected infants (p_adj_ < 0.001, Fig. 5k, Extended Data Fig. 7j,k). Memory T_reg_ cell frequencies were higher in RSV-infected infants compared to other groups (p_adj_ < 0.0016, Fig. 5k, Extended Data Fig. 7k). Most CD4^+^ T cell subsets showed comparably increased ISG scores in both infections (Extended Data Fig. 7l, 8a–i).

The most notable epigenetic differences between RSV and SARS-CoV-2 were observed in naive CD4^+^ T cells (Extended Data Fig. 8j). IRF and STAT binding site accessibility was increased in both infections (Extended Data Fig. 8k), while pro-inflammatory AP-1 and NF-κB binding site accessibility was increased in SARS-CoV-2-infected infants (Extended Data Fig. 8k). Naive CD4^+^ T cells from SARS-CoV-2-infected infants showed higher activity for NF-κB pathway genes at both epigenetic and transcriptional levels (p_adj_ < 0.022, Extended Data Fig. 8l–n).

In summary, both RSV and SARS-CoV-2 infections induced interferon responses across all CD4^+^ T cell subsets. RSV-infected infants had higher frequency of CD4^+^ T_EMRA_ and memory T_reg_ cells and reduced NF-κB activity.

### B cells display increased ISGs in both infections

**Figure 6:**
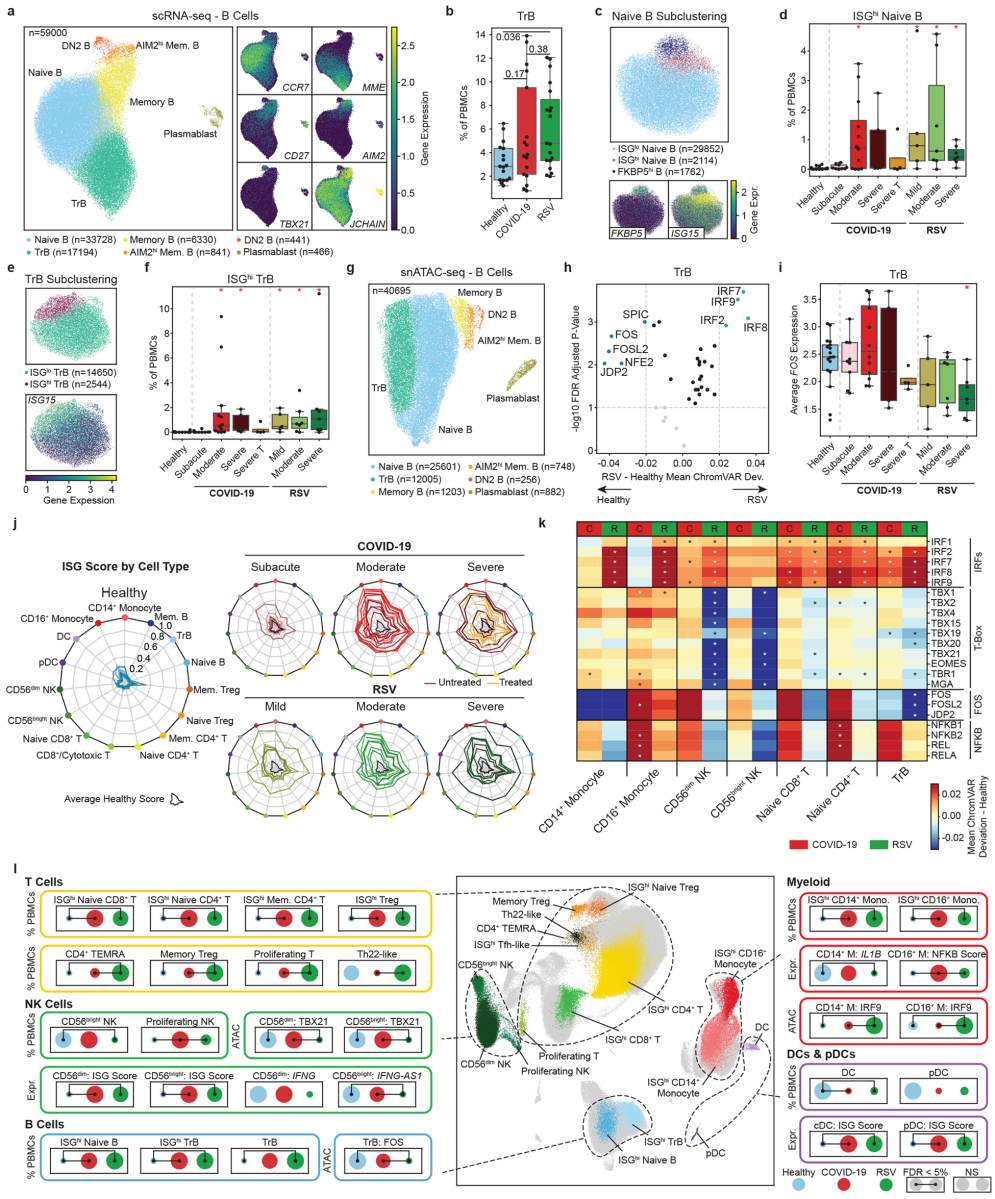
B cell frequencies and summary of SARS-CoV-2 and RSV infections in infants. **a**, UMAP of B cells with marker genes describing naive B (*CCR7*), TrB (*MME*), memory B (*CD27*), AIM2^hi^ memory B (*AIM2*), DN2 (*TBX21*) and plasmablast (*JCHAIN*) cells. **b**, Pairwise comparison of TrB cell frequencies. Adjusted p-values were calculated using Dunn’s test for multiple comparisons. **c**, UMAP of naive B cell clusters describing ISG^lo^ and ISG^hi^ (*ISG15*) naive B, and FKB5hi B (*FKBP5*) cells. **d**, Healthy control comparisons of ISG^hi^ naive B cell frequencies. **e**, UMAP of TrB cell clusters describing ISG^lo^ and ISG^hi^ (*ISG15*) TrB cells. **f**, Healthy control comparisons of ISG^hi^ TrB cell frequencies. **g**, UMAP of snATAC- seq B cell clusters annotated by scRNA-seq *via* label transfer. **h**, Volcano plot of significant transcription factors comparing RSV (mild, moderate and severe) and healthy controls in TrB cells. **i**, Healthy control comparisons of *FOS* expression in TrB cells. **j**, Summary of ISG scores (expression) across PBMC subsets identified in this study for each clinical group. **k**, Summary of chromVAR deviation scores for IRF, T-box, FOS, and NFKB related factors in CD14^+^ monocytes, CD16^+^ monocytes, CD56^dim^ NK, CD56^bright^ NK, naive CD8^+^ T, naive CD4^+^ T and TrB subsets. Asterisks denote significant comparisons (p_adj_ < 0.05) versus healthy controls using Dunn’s test for multiple comparisons. **l**, Highlights of major findings in this study. Dot plots represent the min-max average within healthy controls, COVID-19 (moderate and severe untreated), and RSV (mild, moderate and severe). Lines connecting dots represent significant comparisons using Dunn’s test for multiple comparisons. Statistical tests for healthy control comparisons across clinical groups were calculated using Mann-Whitney rank sum tests followed by Benjamini Hochberg multiple hypothesis correction (panels **d**,**f**,**i**). Significant comparisons (p_adj_ < 0.05) to healthy controls are indicated by a red asterisk.

B cells were clustered into six subsets: naive B (*CCR7*, n = 33,728), transitional B (TrB) (*MME*, n = 17,194), memory B (*CD27*, n = 6,330), AIM2^hi^ memory B (*AIM2*, n = 841), plasmablasts (*JCHAIN*, n = 466) and double-negative-2 (DN2) B (n = 441) cells (Fig. 6a). The frequency of TrB cells was higher in RSV-infected infants (p_adj_ = 0.036, Fig. 6b). No other cell compositional differences were observed across B cell subsets (Extended Data Fig. 9a). Clustering of naive B and TrB cells identified ISG^hi^ subsets, both of which were increased in infected infants (p_adj_ < 0.0037) (Fig. 6c–f, Extended Data Fig. 9b,c). ISG scores were higher across all B cell subsets in infected infants (p_adj_ < 0.05, Extended Data Fig. 9d–i). Epigenetic differences between RSV and SARS-CoV-2 were observed in TrB cells (Fig. 6g,h). In RSV-infected infants, pro-inflammatory AP-1 TFs had reduced chromatin and expression levels (Fig. 6h,i Extended Data Fig. 9j,k).

A subset of naïve B cells highly expressed *FKBP5* (Fig. 6c, Extended Data Fig. 9c), which encodes the FK506-binding protein 51 that interacts with glucocorticoid receptors^29^. The majority (88%) of cells in this cluster originated from steroid-treated COVID-19 infants (n = 4; Extended Data Fig. 9l), suggesting a drug-induced effect on B cells. This FKBP5^hi^ B cell cluster exhibited lower interferon responses (Extended Data Fig. 9m). Comparisons between severe and severe T COVID-19 groups revealed 493 differentially expressed genes and 291 differentially accessible peaks across seven immune cell subsets (Extended Data Fig. 10a, Table S4,5). Some genes, such as *FKBP5*, were consistently upregulated across multiple immune cell subsets, while others, such as *CD163* and *IL1R2* in monocytes, were induced in a cell-type-specific manner (Extended Data Fig. 10b).

In summary, B cells mounted strong interferon responses during RSV and SARS-CoV-2 infections. TrB cells were particularly affected in RSV, showing increased frequencies along with reduced accessibility and expression of AP-1 transcription factors.

## DISCUSSION

This study provides comprehensive multi-modal insights into the immune responses of infants to SARS-COV-2 and RSV infections, revealing both shared and virus-specific immune signatures. Both viruses induced robust interferon responses across PBMC subsets, a hallmark of antiviral immunity, evidenced by the upregulation of interferon-stimulated genes (Fig. 6j) and increased chromatin accessibility at interferon regulatory factor (IRF) binding sites (Fig. 6k). However, we observed key differences in i) inflammatory responses, ii) epigenetic remodeling, and iii) NK and iv) adaptive cell subsets that might underlie the divergent clinical outcomes of these infections (Fig. 6l).

Inflammation emerged as a critical point of divergence between the two infections. SARS-CoV-2 induced strong pro-inflammatory responses, characterized by elevated serum concentrations of TNF, IL-6, and IL-8, alongside upregulated NF-κB pathway activity in monocytes, NK cells, and T cells. These findings are consistent with the acute inflammatory response observed in severe COVID-19 cases^30^. In contrast, RSV infections were associated with reduced expression and chromatin accessibility of *IL1B* in CD14^+^ monocytes, particularly in severe cases, and lower serum concentrations of TNF, IL6, IL8 in serum, indicating a more tempered inflammatory response. Previous findings from our group demonstrated an inverse correlation between innate immunity cytokine levels in nasal wash and plasma and RSV disease severity^18,19,31^. Moreover, infants with severe RSV exhibited increased numbers of HLA-DR^low^ monocytes^12^, often associated with immune suppression. Our findings further revealed transcriptional and epigenetic impairments in circulating monocytes in severe RSV cases, underscoring the critical role of robust innate immune responses in mitigating RSV severity.

NK cell dysregulation was a hallmark of severe RSV infection. The reduction in NK cell frequencies, coupled with impaired *IFNG* expression and diminished chromatin accessibility at T-BET and EOMES binding sites (Fig. 6k,l). NK cell recruitment to the lungs during severe RSV infection has been previously reported^32^, potentially contributing to their depletion in peripheral blood. While reduced *IFNG* expression in total PBMCs has been previously reported in infants with severe RSV^33^, the cellular source of this reduction remained unclear until now.

Epigenetic analyses revealed ISG signatures in monocytes in both infections, including subacute COVID-19 cases, suggesting a durable epigenetic memory of interferon responses. This is consistent with adult studies demonstrating persistent chromatin remodeling months after SARS-CoV-2 infection^21,34^. Notably, the ISGs epigenetically activated in infants mirrored those observed in adults recovering from COVID-19 and were correlated with disease severity. In RSV-infected infants, increased IRF binding site accessibility in monocytes pointed to a distinct regulatory mechanism potentially modulated by viral factors affecting IRF binding site dynamics. These epigenetic changes in RSV-infected infants might help explain the virus’s long term respiratory morbidity (e.g., wheezing and asthma)^35–37^. These findings underscore the critical role of epigenetics in shaping immune responses and infant immunity.

Adaptive immune responses also differed between the infections. RSV infection was associated with increases in CD4^+^ TEMRA cells,, which are expanded in other infectious diseases such as Dengue virus and HIV^38,39^, where they contribute to the elimination of infected cells. Although CD4^+^ TEMRA cells primarily function as effector cells, they can exhibit immunosuppressive properties in chronic infections like HIV^39^. In RSV, the concurrent expansion of memory Tregs and CD4^+^ TEMRA cells may reflect the virus’s capacity to induce immune suppression. The increase in TrB cells in RSV infection may represent a compensatory mechanism for the loss of circulating B cells, as previously observed in RSV cases^11^. In contrast, SARS-CoV-2 infections were characterized by heightened pro-inflammatory transcription factor activity in naive CD4^+^ and CD8^+^ T cells, consistent with the virus’s stronger inflammatory profile.

In severe COVID-19 cases treated with dexamethasone, distinct transcriptional and epigenetic signatures were observed across multiple immune cell types, reflecting the modulatory effects of corticosteroids in infants, similar to findings in adults^16,17^. Dexamethasone-treated infants exhibited reduced expression of NF-κB pathway genes and *IL1B*, consistent with the drug's anti-inflammatory effects. Simultaneously, these infants displayed upregulation of anti-inflammatory markers such as *CD163* and *IL1R2*. Notably, while interferon responses were dampened, they were not entirely suppressed, indicating a complex interplay between inflammation control and antiviral defense.

While this study demonstrates the utility of advanced single-cell and multi-omics approaches in uncovering nuanced immune responses of infants to viral infections, it is limited by its cross-sectional design and relatively small sample sizes for certain clinical groups. Longitudinal studies are needed to evaluate the persistence and functional consequences of the observed transcriptional and epigenetic changes. Future research should incorporate respiratory tract samples to better understand localized immune responses at the primary site of infection. However, repeated invasive sampling in this age group presents significant challenges.

This study highlights the distinct immune responses of infants to SARS-CoV-2 and RSV infections, driven by differences in inflammation, NK cell responses, and epigenetic regulation. These findings deepen our understanding of viral pathogenesis in infants and pave the way for guided clinical decision making to mitigate the burden of these infections in this vulnerable population.

## METHODS

### Study Design

#### SARS-CoV-2 and RSV Cohorts

A convenience sample of children < 2 years of age hospitalized with COVID-19 or respiratory syncytial virus (RSV) infection were prospectively enrolled at Nationwide Children’s Hospital (NCH) in Columbus, Ohio, USA from February 2019 through January 2022. Blood (2.5–5 mL) and nasopharyngeal samples were obtained within 24 hours of hospitalization for multiomic analyses and SARS-CoV-2 and RSV quantitation by real-time polymerase chain reaction (PCR)^40^. During the study period all children hospitalized at NCH, irrespective of the presence of symptoms, underwent nasopharyngeal SARS-CoV-2 testing using a PCR assay per standard of care as described^41^. For RSV infants, testing was performed at the discretion of the attending physician via multiplex PCR panel, and children were excluded if they had any underlying conditions (i.e prematurity, congenital heart disease, chronic lung disease) or use of immunomodulatory drugs including systemic steroids > 5 days within 2 weeks of presentation. At enrollment, we collected demographic and clinical information using a standardized questionnaire designed for the study, and information transferred to a secure database (REDCap). The information collected included duration of illness or time since exposure to an index case, and standard parameters of disease severity including oxygen administration, pediatric intensive care unit (PICU) admission, duration of hospitalization, and administration of systemic therapies including steroids. In addition, we used a standardized clinical disease severity score (CDSS) for infants with RSV infection as previously reported^31,40^. This score was not applied to infants with COVID-19 due to their differing clinical presentations.

### Healthy Controls

As a refence for all immune assays, we also included in the study a cohort of age-matched healthy control infants with no respiratory symptoms or treated with antibiotics within two weeks of enrollment. All healthy controls were enrolled pre-pandemic. Healthy controls were typically enrolled in the operating room while undergoing minor scheduled surgical procedures not involving the respiratory tract, or at the primary care offices during well-child visits.

This study was approved by the Nationwide Children’s Hospital (NCH) IRB (18–00591). Written informed consent was obtained from all children’s guardians before study participation. Further clinical details of all participants are summarized in Table S1.

### PCR Assays for SARS-CoV-2 Viral loads

Nasopharyngeal (NP) swabs were collected at enrollment, placed in viral transport media, transported immediately to the laboratory, aliquoted and stored at − 80°C. Similarly, blood samples were collected in EDTA tubes (BD Vacutainer, Franklin Lakes, NJ, USA), centrifuged, and aliquots of plasma were stored at - 80°C until processed in batches. Viral RNA was extracted from 200 microliters of NP or plasma samples using the QIAcube HT instrument (Qiagen Inc, Germantown, MD, USA) and eluted into 100 microliter volume. In brief, SARS-CoV-2 viral loads were measured using a two-step reverse-transcription (RT) quantitative PCR assay targeting the conserved region of the N1 gene as described^41^. SV loads were measured by quantitative real-time PCR targeting the N gene, as described^31^. Standards and negative controls were included and tested with each PCR assay.

### Cytokine Processing

A panel of 92 cytokines was analyzed with Olink at the Olink Boston Laboratory for 54 samples (n = 37 from this cohort, n = 17 additional healthy controls) over two separate runs. Bridge normalization from OlinkAnalyze R package^42^ was applied to adjust values for comparisons between runs.

### Sample processing for scRNA-seq

PBMCs were thawed quickly at 37°C and into DMEM supplemented with 10% FBS. Cells were quickly spun down at 400 g, for 10 min. Cells were washed once with 1 x PBS supplemented with 0.04% BSA and finally re-suspended in 1 x PBS with 0.04% BSA. Viability was determined using trypan blue staining and measured on a Countess FLII. Briefly, 12,000 cells were loaded for capture onto the Chromium platform using the single cell 3’ gene expression reagent kit (v3 or v3.1) (10x Genomics). Following capture and lysis via Chromium Chip G, cDNA was synthesized and amplified (12 cycles) as per manufacturer's protocol (10x Genomics, protocols CG000204; CG000315). Amplified cDNA and libraries were checked for quality on Agilent 4200 Tapestation, quantified by KAPA qPCR, and sequenced on an Illumina NovaSeq 6000 targeting 100,000 raw read pairs per cell.

### Sample processing for snATAC-seq

For single nucleus ATAC sequencing (snATAC-seq) experiments, viable single cell suspensions from each sample were used to generate snATAC-seq data using the 10x Chromium platform according to the manufacturer’s protocols (10x Genomics, protocols CG000169; CG000168). Briefly, > 100,000 cells from each sample were centrifuged and the supernatant was removed without disrupting the cell pellet. Lysis Buffer was added for 5 minutes on ice to generate isolated and permeabilized nuclei, and the lysis reaction was quenched by dilution with Wash Buffer. After centrifugation to collect the washed nuclei, diluted Nuclei Buffer was used to re-suspend nuclei at the desired nuclei concentration as determined using a Countess II FL Automated Cell Counter and combined with ATAC Buffer and ATAC Enzyme to form a Transposition Mix. Transposed nuclei were immediately combined with Barcoding Reagent, Reducing Agent B and Barcoding Enzyme and loaded onto a 10x Chromium Chip H for droplet generation followed by library construction. The barcoded sequencing libraries were subjected to bead clean-up and checked for quality on an Agilent 4200 TapeStation, quantified by KAPA qPCR, and pooled for sequencing on an Illumina NovaSeq 6000 (2x50bp libraries).

### scRNA-seq data processing

Reads from scRNA-seq were aligned (10x Genomics GRCh38 reference 2020-A) and processed using Cell Ranger v6.1.2^43^. Ambient RNA corrected matrices were obtained by applying SoupX version 1.6.2^44^. Multiplets were removed using Scrublet version 0.2.3^45^ with the following parameters: Doublet Rate = 0.06, Min Counts = 2, Min Cells = 3, Min Gene Variability PCTL = 85, Number of PCs = 30. High-quality cells from each sample were selected using the following selection criteria: 1) mitochondrial percentage: > 1%; < 20%, 2) molecules detected: > 2000; < 25000, and 3) number of genes detected: ≥ 500, resulting in a total of 389,281 high-quality cells.

In addition to the above cell selection protocols, we separated low and high quality cells by applying unsupervised clustering on the following features: 1) number of molecules, 2) number of genes detected, 3) mitochondria read percentage, 4) ribosomal gene percentage, 5) multiplet score (Scrublet) and 6) multiplet annotation (Scrublet). All cells were clustered by applying Uniform Manifold Approximation and Projection (UMAP) followed by clustering *via* HDBSCAN^46^. Using this approach, 3 clusters were associated with low quality or muliplet cells, overlapping 99% with previous criteria and covering 22% of all low quality cells identified in the previous step. An additional 53 low quality cells were for identified, resulting in 389,228 high-quality cells (2000–9161 per sample) before manual filtering.

Ambient mRNA corrected count matrices filtered for the remaining high quality cells from each sample were log normalized in Scanpy (version 1.9.1)^47^ using *normalize_total(target_sum = 1e4)* and *log1p* functions. Scanpy objects from each sample were concatenated into a single object for further processing. Low expressed genes were filtered using the *filter_genes(min_cells = 5)* and highly variable genes were identified using *highly_variable_genes*(). Highly variable genes were scaled using *scale(max_value = 10)* for Principle Component Analysis (PCA): *PCA(svd_solver=‘arpack’, n_comps =* 5*0)*. Principle components were then adjusted for batch effects by applying Harmony^48^ using *harmony_integrate()* in Scanpy. Batch corrected PC values were then projected onto UMAP space and clustered using the Leiden clustering in *Scanpy*.

### scRNA-seq cluster annotation

Cells from scRNA-seq were manually curated and annotated using two rounds of clustering and annotation. In each round, cells were divided into major immune cell lineages (myeloid, B cell, CD4^+^ T Cell, and NK/CD8^+^ and cytotoxic T cells) which were further clustered depending on the granularity required to describe known PBMC subsets. The number of components (adjusted PCA values from harmony) and resolution (Leiden) of clusters were adjusted as necessary depending on the detection of clusters representing known subsets. Clusters were excluded based on the presence of markers from multiple PBMC subsets (i.e. multiplets), red blood cell markers, or high mitochondrial gene percentages. The majority of cells were filtered in the first round, which was focused on identifying clusters for exclusion using the criteria described. Cells remaining after the first round of clustering were re-clustered by reapplying PCA (components = 100) and Harmony for a second round of curation and annotation. In the second round, any remaining multiplet clusters were excluded, and cells were annotated based on known marker genes of known immune cell types. Clusters showing high *ISG15* expression were annotated as ISG^hi^. For CD14^+^ and CD16^+^ monocytes, ISG^hi^ clusters were selected based on scaled *ISG15* expression > 1.

### Memory CD4^+^ T Cell Annotation

Memory CD4^+^ T cells were selected an processed using Seurat (version 5.1.0). Highly variable genes were selected with the FindVariableFeatures function using the following parameters: *selection.method=“vst”* and *nfeatures = 2000*. RunPCA was applied and batch corrected using Harmony using the first 50 components. Clusters were obtained using Leiden with resolution = 1. Density plots were generated using Nebulosa (version 1.14.0)^49^ for known memory CD4 + T markers for annotations.

### Cell frequency comparisons

Cell frequencies were calculated as a percentage of cells within the subset out of the total number of PBMCs in the sample. Comparisons between Healthy, COVID-19 (moderate and severe), and RSV (mild, moderate and severe) were calculated using Dunn’s multiple comparisons test for all pairwise comparisons. Comparisons between clinical groups were calculated using Mann Whitney rank-sum tests for each group against healthy controls followed by Benjamini/Hochberg FDR correction for multiple comparisons.

### Differential gene expression analysis

Aggregated raw gene expression count matrices were obtained for each annotated subset, merging ISG^hi^ and ISG^lo^ subsets and related small subsets (e.g., cDC1s with cDC2s etc) as described in Table S5. CD163^hi^ monocytes resembled CD14^+^ monocytes and were merged with CD14^+^ monocytes to capture differences between severe and severe T COVID-19 groups. For each comparison, samples with fewer than 25 cells in the respective population were excluded. Genes were excluded if fewer than 3 samples had expression in greater than 20% of cells. Sample and gene filtered count matrices were then normalized using *filterByExpr* and *calcNormFactors* functions in EdgeR (version 3.36.0)^50–52^. Differential expression analyses were performed using *estimateDisp* and *glmQLFTest* functions, using batch, sex, and age as covariates. All pairwise comparisons between clinical group as well as combined Healthy, COVID-19 (moderate and severe), and RSV (mild, moderate and severe) comparisons were performed for each annotated subset that had enough samples remaining after filtering to run EdgeR.

### Module analysis

Module enrichments were obtained using genes sets obtained from Altman et al^53^. Briefly, genes were separated into upregulated and downregulated categories based on differential expression analyses. Overlaps with gene modules were tested for statistical significance using Fisher’s Exact Test (one tailed test, alternative hypothesis = greater), and corresponding p-values were corrected for multiple comparisons using Benjamini/Hochberg procedure.

### Gene expression scoring

All gene expression scores were calculated from the h5ad scanpy object as the average expression of all genes in a provided list using the following function in python: *obj.raw.X[:,obj.raw.var.index.isin(genes)].mean(1))*

The following scores were calculated using this method:

*ISG score -* M8.3:Type 1 Interferon module genes from Altman et al^53^.

*NFKB Pathway Score -* BioCarta NFKB Pathway genes obtained from GSEA MSigDB^54^.

Full gene lists are provided in Table S4.

### Coexpression analysis

Cells co-expressing *IL1B* and *ISG15* were selected based on *IL1B* expression > 0.5 and *ISG15* expression > 1.487142. The threshold for *ISG15* was determined by the 75% quantile of all healthy control cells within CD14^+^ monocytes, CD16^+^ monocytes, and DCs. Cells were annotated as positive for one or both genes based on whether their expression exceeded these thresholds.

### snATAC-seq data processing

snATAC-seq reads were aligned (10x Genomics GRCh38 reference 2020-A) and processed using Cell Ranger ATAC v2.1.0^55^. High quality single nucleus data was selected based on the following criteria: percent of reads within exclusion list regions < 5%, nucleosome signal < 4, percent of fragments in peaks > 15%, number of peak region fragments > 3000, percent fragments at transcription start sites > 10%, and percent mitochondrial read fragments < 10%.

Multiplets were identified by applying AMULET^56^ on each sample independently using FDR < 5%. Multiplets were excluded in downstream analyses to better identify multiplet clusters containing multiplets not captured by AMULET. All multiplets identified by AMULET were excluded after first pass filtering to account for homotypic multiplets.

To define a unified set of peaks (genomic regions) for clustering, individual sample peaks from Cell Ranger ATAC were combined by merging peaks if they overlapped by 1 base pair. Peaks with lengths < 20bp; > 10,000bp, falling on chromosome Y, or represented by fewer than 4 samples were excluded from further analysis. Read count matrices were generated from the remaining peaks for each sample.

Read count matrices were concatenated and normalized by applying a python reimplementation of the term frequency inverse document frequency (TF-IDF) normalization method in Signac^57^. Scanpy objects were generated using this matrix, and singular value decomposition (SVD) of the combined TF-IDF normalized matrix was performed using the *pca* function in Scanpy^47^ with *zero_center = False* to perform truncated SVD.

To measure read counts at transcription start sites (TSS) and gene bodies, gene activity scores were computed using a python reimplementation of gene activity score calculations in Signac^57^. Transcription start site and transcription termination sites from UCSC hg38 Refflat database^58^ were used as gene references. Quantifications included reads within the gene body and 2000 base pairs upstream from the transcription start sites.

### snATAC-seq label transfer from scRNA-seq

Nuclei/cells from snATAC-seq and scRNA-seq were divided into 4 lineages: 1) myeloid, 2) CD4^+^ T, 3) NK, CD8^+^ and cytotoxic T, and 4) B cells based on gene activity scores and gene expression values respectively. Within each lineage, cluster annotations from scRNA-seq were transferred onto snATAC- seq cells using *FindTransferAnchors* with *reduction=’CCA’* and *TransferData* functions in Signac (version 1.7.0)^57^ and Seurat (version 4.1.1)^59^. Nuclei were then assigned scRNA-seq annotations based on highest probability scores. Based on these annotations, clusters in snATAC-seq data were assigned to reflect the majority of cell predictions within the cluster.

### Differential accessibility analysis

Aggregated raw read count matrices were obtained for each annotated subset using peaks called for the respective subset to define the genomic regions used in these matrices. Small subsets were merged (e.g., cDC1s with cDC2s etc) as necessary to increase cell numbers. For each comparison, samples with fewer than 50 cells in the respective population were excluded. Peaks were excluded if fewer than 3 samples had reads > 0. Sample and gene filtered count matrices were then normalized using *filterByExpr* and *calcNormFactors* functions in EdgeR (version 3.36.0)^50–52^. Differential accessibility analyses were performed using *estimateDisp* and *glmQLFTest* functions, using batch, sex, and age as covariates. All pairwise comparisons between clinical groups as well as combined Healthy, COVID-19 (moderate and severe), and RSV (mild, moderate and severe) comparisons were performed for each annotated subset that had enough samples remaining after filtering to run EdgeR.

### Nearest expressed gene identification

Transcription start sites from UCSC hg38 Refflat database^58^ (Downloaded April 2023) were used for identifying nearest expressed gene targets. TSS positions were excluded if corresponding genes did not have expression values > 0 for any sample within a given PBMC subset.

### Epigenetic ISG scoring

Differentially accessible peaks in CD14^+^ or CD16^+^ monocytes were selected based on if they were opening with infection and if their nearest expressed gene was among interferon modules from Altman et al^53^: M10.1:Interferon, M13.17:Interferon, M8.3:Type 1 Interferon, M15.127:Interferon, M15.64:Interferon, M15.86:Interferon. Epigenetic ISG scores were calculated as the average batch corrected log2 counts per million (CPM) for the selected peaks.

### Epigenetic ISG scoring in convalescent COVID-19 adults

Adult healthy, mild convalescent COVID-19 and severe convalescent COVID-19 CD14^+^ monocyte paired end ATAC-seq reads from Cheong et al^21^ (GSE196990) were processed using the ATAC-seq pipeline available at https://github.com/UcarLab/ATAC-seq. Epigenetic ISG scores were then calculated as the average log2 CPM counts using the same regions for epigenetic ISG scores identified in infants.

### NFKB ChromVAR Score:

NFKB ChromVAR scores were calculated as the mean of the following ChromVAR deviation scores: MA0105.4_NFKB1, MA0778.1_NFKB2, MA0101.1_REL and MA0107.1_RELA.

### Domains of opening chromatin (DORC)

Cells between scRNA-seq and snATAC-seq from the same sample were paired using the *pairCells* function in FigR^60^ version 0.1.0 with the following parameters: *keepUnique = True*, *max_multimatch = 5*, *min_subgraph_size = 60*. The *search_range* parameter was adjusted between 0.05–0.25 as necessary for the function to successfully finish, taking the pairing with the highest search_range as the final pairing. Cell pairings were then filtered by cell annotations such that pairs with consistent cell type annotations between scRNA-seq and snATAC-seq were kept and all other pairs were excluded. Using inferred cell pairings, DORC scores were obtained with FigR by applying *runGenePeakcorr*, *dorcJPlot*, and *getDORCScores* methods and using *pvalZ < 0.05* and *cutoff = 8* to filter correlations and DORCs.

### Transcription factor motif binding site accessibility

Transcription factor motif binding sites accessibility deviations were calculated using chromVAR version 1.16.0^23^. For each PBMC subset, genomic regions for chromVAR analysis were selected by extending MACS2 summit locations ± 250 base pairs (bp). Read count matrices were then generated based on single nucleus read counts within these regions. TF motif deviations were then calculated using *computeDeviations* in chromVAR, using motifs from *getJasparMotifs()*, (CORE set JASPAR 2016^61^, homo sapiens).

Motifs were selected based on TFs with expression > 0 within the respective PBMC subset. Kruskal-Wallis test was applied on median motif deviation scores for each sample across clinical groups and p-values were adjusted for multiple comparisons within each PBMC subset using Benjamini/Hochberg procedure. Finally, significant motifs were selected using p_adj_ values < 0.1.

### Motif detection

The *annotatePeaks.pl* function in HOMER version 4.11.1^62^ was used to find instances of motifs within peaks. The findMotifsGenome.pl function in HOMER was used for determining motifs enriched for specific genomic regions. In both analyses, JASPAR-vertebrate (2020)^63^ motifs filtered for homo sapiens were used as known motifs. Detection thresholds for position weight matrices (PWMs) used in HOMER were calculated as half of the score for perfect matching: $\sum \frac{\log\left(\frac{\max\left(p\right)}{0.25}\right)}{2}$ where p is the probability for one nucleotide in a motif.

### Log2 CPM matrix Batch Correction

Log2 counts per million (CPM) matrices for differential expression and differential accessibility analysis were corrected with the *ComBat* function of SVA version 3.51.0^64^, using batch as a covariate.

### Use of large language models

Large language models including ChatGPT and Copilot were used for writing assistance including grammar check and revising text written by authors.

## Supplementary Material

Supplement 1

## Data Availability

Data can be viewed at the following: https://covidrsvinfants.jax.org. Processed data is available on GEO: GSE283746 (token: uzkvsmymppqjtmv) for scRNA-seq, GSE283744 (token: wdkhaccizpmltgn) for snATAC-seq. Raw read data will be made available on dbGAP upon acceptance of the manuscript.
