# Supplementary material for: Infants display reduced NK cell responses in RSV and increased inflammatory responses in SARS-CoV-2 infections
Source: Res Sq. 2025 Jan 13:rs.3.rs-5640872. Preprint. [Version 1] doi: 10.21203/rs.3.rs-5640872/v1 (PMC11774461; doi:10.21203/rs.3.rs-5640872/v1)
Supplement: Supplement 1 [file NIHPPrs5640872v1-supplement-1.pdf]

Supplementary Files

This is a list of supplementary files associated with this preprint. Click to download.

- ExtendedDataFigs.docx
- TableS1SampleData.xlsx
- TableS2CellCompositionData.xlsx
- TableS3CytokineData.xlsx
- TableS4Scoring.xlsx
- TableS5DESummary.xlsx
- TableS6DASummary.xlsx
- TableS7DORC.xlsx
- TableS8ChromVAR.xlsx
